# TCRpred: incorporating T-cell receptor repertoire for clinical outcome prediction

**DOI:** 10.3389/fgene.2024.1345559

**Published:** 2024-03-13

**Authors:** Meiling Liu, Yang Liu, Li Hsu, Qianchuan He

**Affiliations:** ^1^ Public Health Sciences Division, Fred Hutchinson Cancer Center, Seattle, WA, United States; ^2^ Department of Mathematics and Statistics, Wright State University, Dayton, OH, United States

**Keywords:** CDR3, clinical outcome prediction, high dimensions, high throughput sequencing, T-cell receptor, TCR repertoire

## Abstract

T-cell receptor (TCR) plays critical roles in recognizing antigen peptides and mediating adaptive immune response against disease. High-throughput technologies have enabled the sequencing of TCR repertoire at the single nucleotide level, allowing researchers to characterize TCR sequences with high resolutions. The TCR sequences provide important information about patients’ adaptive immune system, and have the potential to improve clinical outcome prediction. However, it is challenging to incorporate the TCR repertoire data for prediction, because the data is unstructured, highly complex, and TCR sequences vary widely in their compositions and abundances across different individuals. We introduce TCRpred, an analytic tool for incorporating TCR repertoire for clinical outcome prediction. The TCRpred is able to utilize features that can be extracted from the TCR amino acid sequences, as well as features that are hidden in the TCR amino acid sequences and are hard to extract. Simulation studies show that the proposed approach has a good performance in predicting clinical outcome and tends to be more powerful than potential alternative approaches. We apply the TCRpred to real cancer datasets and demonstrate its practical utility in clinical outcome prediction.

## 1 Introduction

T cell is one of the most important components of the adaptive immune system and plays fundamental roles in fighting diseases (Kumar et al., 2018). The functions of T cells critically depend on the T-cell receptor (TCR), a protein complex that is expressed on the surface of T cells and can recognize an astronomical number of antigens from pathogens or tumor cells (Chopp et al., 2022). Recent research has shown that TCR repertoire can be indicative of the functional activity of tumor infiltrating T cells and predict disease course in cancer progression (Valpione et al., 2021; Shafer et al., 2022). Indeed, sequencing the TCR repertoire and utilizing the sequence information for clinical outcome prediction have become a vital task in cancer research.

The TCR complex is a transmembrane heterodimer linked by disulfide bonds. In humans, about 95% of T cells are comprised of the alpha and beta chains, and the remaining 5% of T cells consist of the gamma and delta chains (Shah et al., 2021). For the TCR alpha chain, its diversity is mainly generated by the random rearrangement of the variable (V) and the joining (J) gene segments, while for the beta chain, the random rearrangement involves the V and J segments plus the diversity (D) gene segment (Tanno et al., 2020). Due to the extra D segment, the beta chain is more diverse than the alpha chain, and thus we focused on the beta chain. Within the beta chain, a region of particular interest is the complementarity-determining region 3 (CDR3), which is generally considered to be the principal binding site for antigens (Rock et al., 1994). In this article, we focus on the CDR3 of the TCR beta chain for risk prediction.

It is challenging to incorporate TCR repertoire for clinical outcome prediction because TCR repertoires are highly diverse and little overlapped among individuals. In fact, the TCR diversity involves not only the recombination of the V(D)J gene segments, but also the random addition or deletion of nucleotides at the junctions between gene segments. It is estimated that the degree of TCR diversity can reach up to the order of 1 × 10^15^ (Clambey et al., 2014). Meanwhile, TCR repertoires from different individuals generally have distinct profiles, i.e., their TCR compositions and abundances differ substantially. Animal model studies showed that the overlap between TCR repertoires of two genetically identical organisms is around only 20% (Nikolich-Žugich et al., 2004). Thus, TCR repertoire data carry few common features that can be used for clinical outcome prediction. For this reason, a common practice in TCR analysis is to calculate the Shannon entropy for each individual and then use this quantity for risk prediction (Li et al., 2020). However, the Shannon entropy, by its definition, accounts for only the proportions of TCR sequences, while the rich information embedded in the TCR amino-acid sequences is largely neglected. Feature extraction is needed for a more efficient use of the TCR sequences. In addition, some features hidden in the TCR sequences may be difficult to extract due to the complexity of the structure and functions of the TCR. Examples include structural motifs, 3D conformations, and amino acid interactions (Parras-Moltó et al., 2013; Stiffler et al., 2020). It is desirable to incorporate these hidden features in risk modeling to potentially improve the prediction accuracy.

Recently, a number of tools have been developed for studying the TCR repertoire, such as the powerTCR (Desponds et al., 2016), Immunarch (ImmunoMind Team, 2019), ImSpectR (Cordes et al., 2020), and VisTCR (Ni et al., 2020). These tools provide a variety of functionalities for TCR analysis, ranging from comparing clonal distribution and tracking clonotype to quantifying repertoire diversity and data visualization. Few methods have been developed for predicting clinical outcome of interest using the TCR repertoire information, which is believed to play important roles in immune responses to tumor progression. The TCR-L method (Liu et al., 2022) is for conducting genetic association analysis, not for risk prediction. The DeepTCR (Sidhom et al., 2021) was proposed to utilize TCR repertoire for prediction, but does not accommodate adjusting covariates such as demographic and clinical variables. Here, we propose a powerful approach, TCRpred, for predicting continuous or binary outcome by incorporating TCR repertoire with existing demographic and clinical factors. In TCRpred, the effect of the TCR repertoire is characterized by two components: 1) the effect from features that can be extracted from the TCR sequences, such as amino acid *k*-mers or V(D)J gene usage, and 2) the effect from hidden features that are modeled through kernel machine techniques. Then, we relate the two types of effects (along with other risk factors’ effects) to the clinical outcome through a generalized linear model. An effective algorithm is proposed to optimize the objective function to estimate the regression coefficients, which are then used to predict clinical outcomes for new observations.

Our article is organized as follows. We describe the TCRpred method in detail in the Model and method section. In the Simulation section, we conduct simulation studies under various scenarios to evaluate the performance of the proposed approach and compare it to potential alternative methods. In the Real data analysis section, we apply TCRpred to lung cancer datasets from the Cancer Genome Atlas (TCGA) and show that the TCRpred method performs well in practical data analysis.

## 2 Model and method

### 2.1 Notation and model

Assume that there are *n* individuals in a study. For the *i*th individual, let *Y*_*i*_ be a binary or continuous response, and $X_{i}={\left(X_{i1},\ldots ,X_{ir}\right)}^{T}$ be a vector of *r* adjusting variables, such as age, gender, and lab measurements. Assume that the *i*th individual contains *m*_*i*_ unique amino acid sequences. Among the *m*_*i*_ unique sequences, let *a*_*ij*_ denote the *j*th amino acid sequence, and *w*_*ij*_ be the corresponding abundance of *a*_*ij*_. Then, the TCR repertoire of the *i*th individual can be represented by *R*_*i*_ = {(*a*_*i*,*j*_, *w*_*i*,*j*_); *j* = 1, …, *m*_*i*_}.

Given two individuals (*i* and *i*′), *R*_*i*_ often differs substantially from *R*_*i*’_ in their compositions and abundances, and hence there are few common features that can be directly used for clinical outcome prediction. We propose extracting features from the TCR repertoire based on TCR’s sequence information. Given that each TCR-CDR3 sequence is a string of amino acid letters (such as CASSHGRAEAFF), we consider the strategy of extracting *k*-mers from each sequence and then aggregate the *k*-mers across all the sequences in a TCR repertoire. An example is shown in Supplementary Figure S1. This strategy shares spirit with the natural language processing where *k*-grams, contiguous sequences of *k* items from a given document, are extracted for text classification (Zhang and Rao, 2020). Because the number of amino acids is 20, the number of possible *k*-mers is 20^*k*^, which increases rapidly with *k*. For example, the number of possible 4-mers is 20^4^ = 160, 000. The extremely high dimensionality poses tremendous challenges to data analysis and can potentially harm the prediction accuracy. Hence, in practical analysis of amino acid sequences, *k* is often chosen to be between 2 and 5 (ValizadehAslani et al., 2020). Besides the *k*-mers extraction, other ways to extract features from the TCR sequence, such as counting the V(D)J gene usage, can be adopted as well. Let *Z* (*R*_*i*_) denote the vector of all the features extracted from *R*_*i*_. For ease of notation, we use *Z*_*i*_ to represent *Z* (*R*_*i*_) in the remainder of this article.

**FIGURE 1:**
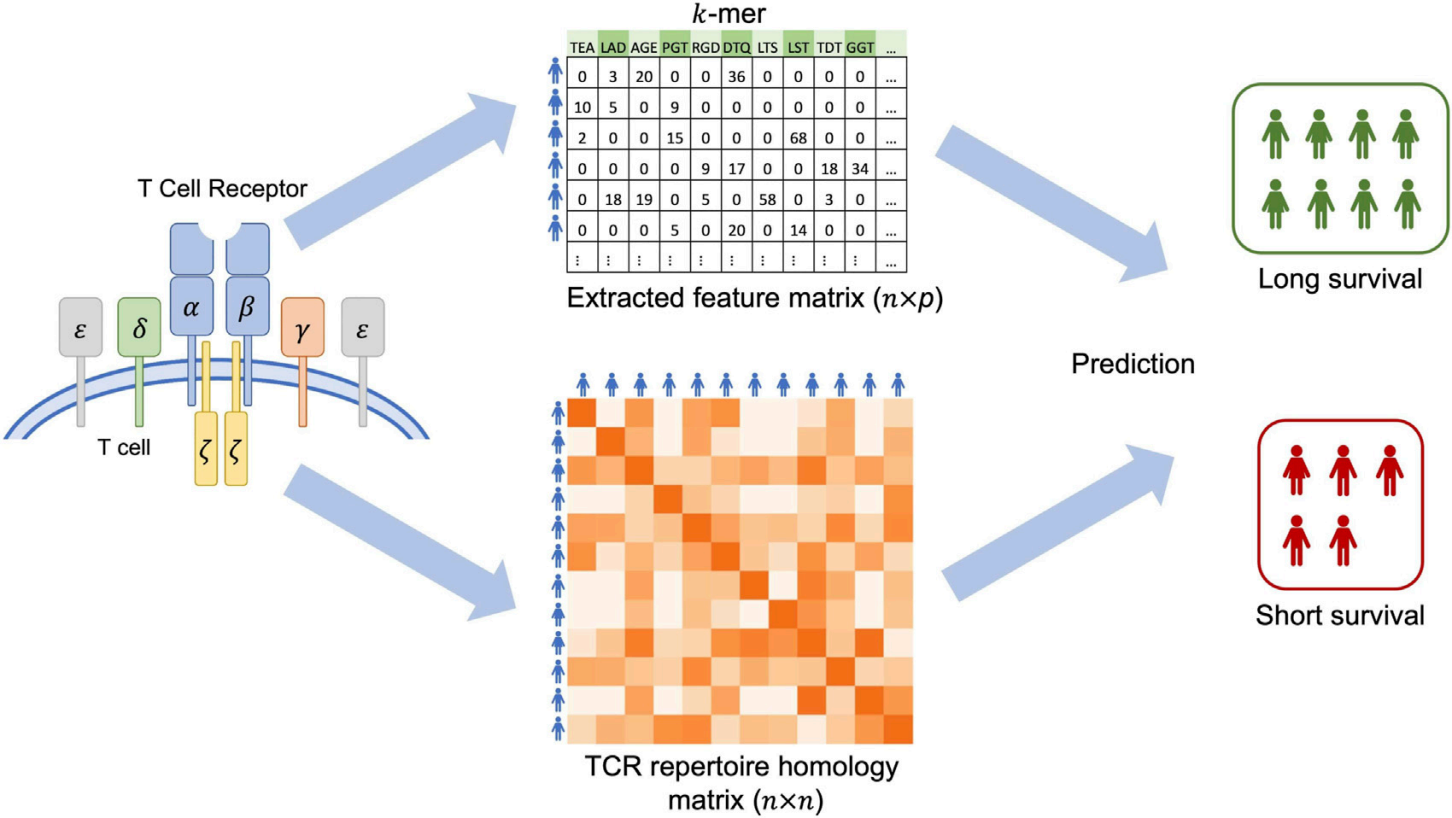
The workflow of the TCRpred.

While some features can be explicitly extracted from the TCR sequences, other features that involve tertiary structure or long-range amino acid interactions are often difficult to extract. To accommodate such hidden features, we consider the following semi-parametric model where the effect of the hidden features is modeled through kernel machines. Let *π*(⋅) denote a link function. For continuous traits, *π*(⋅) is the identity function, and for binary traits, *π*(*x*) = exp(*x*)/(1 + exp(*x*)). Then the mean of *Y*_*i*_ can be represented by

$$\text{E}\left(Y_{i}\right)=\pi \left(\beta_{0}+X_{i}^{T}\beta +Z_{i}^{T}\gamma +h\left(R_{i}\right)\right),$$ (1)

where *β*_0_ is an intercept, *β* and *γ* are regression coefficients, and *h* (*R*_*i*_) represents the effect of the hidden features. Under the kernel machine framework, we assume that *h* (⋅) belongs to a reproducing kernel Hilbert space $H_{K}$ generated by a kernel function *k* (⋅, ⋅). Here, *k* (*R*_*i*_, *R*_*i*’_) measures the homology between individuals *i* and *i*′ based on their TCR repertoires. We adopt the TCRhom approach to calculate *k* (*R*_*i*_, *R*_*i*’_) (Liu et al., 2022). Briefly, let *s* (*a*_*i*,*j*_, *a*_*i*′,*j*′_) be the similarity between two TCR sequences *a*_*i*,*j*_ and *a*_*i*′,*j*′_, where the similarity is calculated based on sequence alignment and a subtitution matrix (such as the BLOSUM62 or PAM250). Then, the homology between two individuals’ TCR repertoires is calculated by

$$k\left(R_{i},R_{i^{\prime }}\right)=\frac{\sum_{j=1}^{m_{i}}w_{i,j}\underset{j^{\prime }\in M_{i^{\prime }}}{\max}s\left(a_{i,j},a_{i^{\prime },j^{\prime }}\right)+\sum_{j^{\prime }=1}^{m_{i^{\prime }}}w_{i^{\prime },j^{\prime }}\underset{j\in M_{i}}{\max}s\left(a_{i,j},a_{i^{\prime },j^{\prime }}\right)}{\sum_{j=1}^{m_{i}}w_{i,j}+\sum_{j^{\prime }=1}^{m_{i}^{\prime }}w_{i^{\prime },j^{\prime }}},$$

where *M*_*i*_ = {1, *…*, *m*_*i*_} and *M*_*i*’_ = {1, *…*, *m*_*i*′_} for *i*, *i*′ = 1, *…*, *n*. Let *K* be an *n* × *n* matrix defined based on *k* (*R*_*i*_, *R*_*i*’_). The *k* (*R*_*i*_, *R*_*i*’_) accounts for both the amino acid information and the abundances of the TCR sequences, and fully characterizes the functional space of the hidden effect *h* (*R*_*i*_). A workflow of the TCRpred is shown in Figure 1.

### 2.2 Continuous outcome

To build the prediction model, we need to estimate parameters in Eq 1 and obtain an explicit form for *h* (⋅). First, considering that the extracted features can be high dimensional, we impose a penalty to the regression coefficients *γ* to reduce dimensions (i.e., remove noise features). Then, under the kernel machine framework, the estimation proceeds by minimizing the following penalized loss function

$$\overset{n}{\underset{i=1}{\sum }}{\left(Y_{i}-\beta_{0}-X_{i}^{T}\beta -Z_{i}^{T}\gamma -h\left(R_{i}\right)\right)}^{2}+\lambda_{0}|\gamma {|}_{1}+\lambda_{1}\|h{\|}_{H_{K}}^{2},$$

where *λ*_0_ and *λ*_1_ are regularization parameters, |⋅|_1_ is the *L*_1_ norm, and $\|\cdot {\|}_{H_{K}}$ is the norm under the generated functional space $H_{K}$ . Here, *λ*_0_|*γ*|_1_ is for conducting variable selection for the extracted features, while $\lambda_{1}\|h{\|}_{H_{K}}^{2}$ is for balancing goodness of fit and complexity of the model.

We propose the following procedure to solve the above optimization problem. Notice that when *β*_0_, *β*, *γ* are fixed, by the Representer’s Theorem, a general solution for *h* (*R*_*i*_) can be expressed as $h\left(R_{i}\right)=K_{i}^{T}\alpha$ , where *K*_*i*_ is the *i*th column of *K*, and *α* is an *n* × 1 vector. Then, the objective function becomes

$$\overset{n}{\underset{i=1}{\sum }}{\left(Y_{i}-\beta_{0}-X_{i}^{T}\beta -Z_{i}^{T}\gamma -K_{i}^{T}\alpha \right)}^{2}+\lambda_{0}|\gamma {|}_{1}+\lambda_{1}\alpha^{T}K\alpha .$$

Let $Y={\left(Y_{1},\ldots ,Y_{n}\right)}^{T},X={\left(X_{1},\ldots ,X_{n}\right)}^{T}$ , and $Z={\left(Z_{1},\ldots ,Z_{n}\right)}^{T}$ . Let *Z* ^(*t*)^ denote the updated version of *Z* in the *t*th iteration. Let *p* ^(*t*)^ denote the number of columns in *Z* ^(*t*)^, and let ${\hat{\beta }}_{0}^{\left(t\right)},{\hat{\beta }}^{\left(t\right)}$ , ${\hat{\gamma }}^{\left(t\right)}$ and ${\hat{\alpha }}^{\left(t\right)}$ denote the coefficient estimates in the *t*th iteration.

For initialization, let *Z* ^(0)^ = *Z* and the starting value ${\hat{\alpha }}^{\left(0\right)}=0$ . For iteration *t* = 1, 2, …, do the following steps:

Step 1. Fix ${\hat{\alpha }}^{\left(t-1\right)}$ , then minimizing the objective function is equivalent to minimizing the following function with respect to *β*_0_, *β*, *γ*:

$$\overset{n}{\underset{i=1}{\sum }}{\left(Y_{i}-K_{i}^{T}{\hat{\alpha }}^{\left(t-1\right)}-\beta_{0}-X_{i}^{T}\beta -Z_{i}^{\left(t-1\right)T}\gamma \right)}^{2}+\lambda_{0}|\gamma {|}_{1}.$$ (2)

By minimizing Eq 2, we obtain estimates ${\hat{\beta }}_{0}^{\left(t\right)},{\hat{\beta }}^{\left(t\right)}$ and ${\hat{\gamma }}^{\left(t\right)}$ . For features in *Z* ^(*t*−1)^, we retain features that have nonzero coefficients in ${\hat{\gamma }}^{\left(t\right)}$ , and then use the retained features to form a new matrix *Z* ^(*t*)^. With slight abuse of notation, the nonzero part of ${\hat{\gamma }}^{\left(t\right)}$ is still named as ${\hat{\gamma }}^{\left(t\right)}$ .

Step 2. Fix ${\hat{\beta }}_{0}^{\left(t\right)},{\hat{\beta }}^{\left(t\right)}$ and ${\hat{\gamma }}^{\left(t\right)}$ , we estimate *α*. Then, the objective function becomes

$$\overset{n}{\underset{i=1}{\sum }}{\left(Y_{i}-{\hat{\beta }}_{0}^{\left(t\right)}-X_{i}^{T}{\hat{\beta }}^{\left(t\right)}-Z_{i}^{\left(t\right)T}{\hat{\gamma }}^{\left(t\right)}-K_{i}^{T}\alpha \right)}^{2}+\lambda_{1}\alpha^{T}K\alpha .$$

Then, we estimate *α* as follows,

$${\hat{\alpha }}^{\left(t\right)}={\left(\lambda_{1}^{\left(t\right)}I+K\right)}^{-1}\left(Y-{\hat{\beta }}_{0}^{\left(t\right)}-X{\hat{\beta }}^{\left(t\right)}-Z^{\left(t\right)}{\hat{\gamma }}^{\left(t\right)}\right),$$

where I is an identity matrix. In line with He et al. (2016), we set $\lambda_{1}^{\left(t\right)}$ as $\sqrt{\left(p^{\left(t\right)}+r+1\right)/n}$ , where *r* is the number of adjusting variables.

Step 3. Compute the error term

$${\hat{e}}^{\left(t\right)}=Y-\left({\hat{\beta }}_{0}^{\left(t\right)}+X{\hat{\beta }}^{\left(t\right)}+Z^{\left(t\right)}{\hat{\gamma }}^{\left(t\right)}+K{\hat{\alpha }}^{\left(t\right)}\right),$$

and then calculate the ${MSE}^{\left(t\right)}={\hat{e}}^{\left(t\right)T}{\hat{e}}^{\left(t\right)}/n$ .

Define MSE^(0)^ to be +*∞*. Iterate Steps 1 - 3 until a convergence criterion is met, i.e., |MSE^(*t*)^ − MSE^(*t*−1)^| ≤ *ϵ* for a small value of *ϵ* or the maximum number of iterations is reached.

Once the model has been trained, we can use the trained model to conduct prediction tasks. Suppose that a new sample consists of *X*_*i*’_ and *R*_*i*’_. We first extract features from *R*_*i*’_. Based on the features in the final *Z* ^(*t*)^, we extract the corresponding features from *R*_*i*’_ to form a feature vector *Z*_*i*’_. Then, we calculate the TCR homology between the new individual and the previous *n* training individuals, yielding a *n* × 1 vector which is denoted by *K*_*i*’_. Then, we plug in *X*_*i*’_, *Z*_*i*’_, and *K*_*i*’_ into the trained model,

$${\hat{\beta }}_{0}^{\left(t\right)}+X_{i^{\prime }}^{T}{\hat{\beta }}^{\left(t\right)}+Z_{i^{\prime }}^{T}{\hat{\gamma }}^{\left(t\right)}+K_{i^{\prime }}^{T}{\hat{\alpha }}^{\left(t\right)},$$

to obtain the predicted value ${\hat{y}}_{i^{\prime }}$ .

### 2.3 Binary outcome

For the binary outcome, we have the objective function as

$$\overset{n}{\underset{i=1}{\sum }}[-Y_{i}\left(\beta_{0}+X_{i}^{T}\beta +Z_{i}^{T}\gamma +h\left(R_{i}\right)\right)+\log\left\{1+\exp\left(\beta_{0}+X_{i}^{T}\beta +Z_{i}^{T}\gamma +h\left(R_{i}\right)\right)\right\}]+\lambda_{2}|\gamma {|}_{1}+\lambda_{3}\|h{\|}_{H_{K}}^{2},$$

where *λ*_2_ and *λ*_3_ are regularization parameters.

As for the linear outcome, we propose an iterated procedure to solve the optimization problem. With a similar argument and by the Representer’s Theorem, we aim to solve the following objective function

$$\overset{n}{\underset{i=1}{\sum }}\left[-Y_{i}\left(\beta_{0}+X_{i}^{T}\beta +Z_{i}^{T}\gamma +K_{i}^{T}\alpha \right)+\log\left\{1+\exp\left(\beta_{0}+X_{i}^{T}\beta +Z_{i}^{T}\gamma +K_{i}^{T}\alpha \right)\right\}\right]+\lambda_{2}|\gamma {|}_{1}+\lambda_{3}\alpha^{T}K\alpha .$$

To minimize this objective function, we propose to transform the binary outcome into a linearized form (Park and Hastie, 2008) and then conduct the optimization accordingly. For initialization, we fit a regularized logistic regression for *Y* with respect to *X* and *Z*, i.e.,

$$\underset{\beta_{0},\beta ,\gamma }{\mathrm{arg min}}\overset{n}{\underset{i=1}{\sum }}\left[-Y_{i}\left(\beta_{0}+X_{i}^{T}\beta +Z_{i}^{T}\gamma \right)+\log\left\{1+\exp\left(\beta_{0}+X_{i}^{T}\beta +Z_{i}^{T}\gamma \right)\right\}\right]+\lambda_{2}|\gamma {|}_{1},$$

to obtain estimates ${\hat{\beta }}_{0}^{\left(0\right)},{\hat{\beta }}^{\left(0\right)}$ and ${\hat{\gamma }}^{\left(0\right)}$ . Let $Y_{w}^{\left(0\right)}=Y,Z^{\left(0\right)}=Z$ , ${\hat{\alpha }}^{\left(0\right)}=0$ , and $\hat{h}{\left(R_{i}\right)}^{\left(0\right)}=0$ . For iteration *t* = 1, 2, …, compute the following:

Step 1. Compute the working response

$$Y_{w,i}^{\left(t\right)}=\Delta_{i}^{\left(t\right)}+\frac{Y_{i}-\pi \left(\Delta_{i}^{\left(t\right)}\right)}{\pi \left(\Delta_{i}^{\left(t\right)}\right)\left(1-\pi \left(\Delta_{i}^{\left(t\right)}\right)\right)},$$

where $\Delta_{i}^{\left(t\right)}={\hat{\beta }}_{0}^{\left(t-1\right)}+X_{i}{\hat{\beta }}^{\left(t-1\right)}+Z_{i}^{\left(t-1\right)}{\hat{\gamma }}^{\left(t-1\right)}+\hat{h}{\left(R_{i}\right)}^{\left(t-1\right)}$ .

Step 2. Let $\nu_{i}^{\left(t\right)}=\pi \left(\Delta_{i}^{\left(t\right)}\right)\left(1-\pi \left(\Delta_{i}^{\left(t\right)}\right)\right)$ , and let Ω^(*t*)^ be a *n* × *n* diagonal matrix with its diagonal elements being $\nu_{i}^{\left(t\right)}$ . Then, conduct the following penalized regression,


$$\underset{\beta_{0},\beta ,\gamma }{\mathrm{arg min}}\overset{n}{\underset{i=1}{\sum }}\nu_{i}^{\left(t\right)}{\left(Y_{w,i}^{\left(t\right)}-K_{i}^{T}{\hat{\alpha }}^{\left(t-1\right)}-\beta_{0}-X_{i}^{T}\beta -Z_{i}^{\left(t-1\right)T}\gamma \right)}^{2}+\lambda_{2}|\gamma {|}_{1},$$

to obtain estimates ${\hat{\beta }}_{0}^{\left(t\right)},{\hat{\beta }}^{\left(t\right)}$ and ${\hat{\gamma }}^{\left(t\right)}$ . For features in *Z* ^(*t*−1)^, the ones that have non-zero coefficient estimates are selected and form a new matrix *Z* ^(*t*)^. Then, the nonzero part of ${\hat{\gamma }}^{\left(t\right)}$ is named as ${\hat{\gamma }}^{\left(t\right)}$ .

Step 3. Following a similar argument to the linear outcome, we estimate *α* by


$${\hat{\alpha }}^{\left(t\right)}={\left(\lambda^{\left(t\right)}I+\Omega^{\left(t\right)1/2}K\right)}^{-1}\Omega^{\left(t\right)1/2}\left(Y_{w}^{\left(t\right)}-{\hat{\beta }}_{0}^{\left(t\right)}-X{\hat{\beta }}^{\left(t\right)}-Z^{\left(t\right)}{\hat{\gamma }}^{\left(t\right)}\right),$$

where $Y_{w}^{\left(t\right)}={\left(Y_{w,1}^{\left(t\right)},\ldots ,Y_{w,n}^{\left(t\right)}\right)}^{T}$ , and $\lambda^{\left(t\right)}=\sqrt{\left(p^{\left(t\right)}+r+1\right)/n}$ . Let $\hat{h}{\left(R_{i}\right)}^{\left(t\right)}=K_{i}^{T}{\hat{\alpha }}^{\left(t\right)}$ .

Step 4. Compute the cross entropy ${\hat{e}}^{\left(t\right)}$ for performance evaluation. Let

$${\hat{e}}^{\left(t\right)}=-\frac{1}{n}\underset{i}{\sum }\left[Y_{i}\log\left(\pi \left(\Delta_{i}^{\left(t\right)}\right)\right)+\left(1-Y_{i}\right)\log\left(1-\pi \left(\Delta_{i}^{\left(t\right)}\right)\right)\right].$$

Iterate Steps 1 - 4 above until a convergence criterion is met, i.e., $|{\hat{e}}^{\left(t\right)}-{\hat{e}}^{\left(t-1\right)}|\leq \epsilon$ for a small value of *ϵ* or the maximum number of iteration is reached.

In practice, because the number of extracted features is ultra high dimensional, one may need to conduct a feature-screening step before applying the above algorithm. To do this, we propose to first exclude *k*-mers whose frequencies are less than 5%. Then, we conduct a sure-independence screening to reduce the dimension of *Z* to a moderate number, e.g., *n*/(2 log  *n*).

## 3 Simulation

In this section, we conducted simulation studies to examine the performance of the proposed approach. We first built a pool of TCR repertoires using the Cancer Genome Atlas (TCGA) data. We extracted TCR beta-chain’s CDR3 sequences from TCGA’s RNA-Seq data following Chen et al. (2021). We removed TCR sequences that had abundance equal to 1 or contained abnormal amino acid letters. Individuals with a single TCR sequence were excluded. After data processing, we obtained 8,044 individuals’ TCR repertoires as a TCR pool for the subsequent numerical experiments.

Following the outline of Eq 1, we simulated an adjusting variable *X*_*i*1_ from *N* (0, 1) for *i* = 1, …, *n*. The intercept and the coefficient for *X*_*i*1_ were set as *β*_0_ = 2, *β*_1_ = −1, respectively. For each individual, we randomly sampled a TCR repertoire, i.e., *R*_*i*_, from the TCGA-TCR pool. We considered *k* = 3, 4 for the binary outcome and *k* = 3, 4, 5 for the continuous outcome. Then, for a given *k*, we extracted all *k*-mers from *R*_*i*_ (*i* = 1, …, *n*), and recorded the frequencies of the *k*-mers as a feature matrix *Z*. Since *Z* may have uneven variances for its columns, we normalized *Z* by dividing each column by its 0.75 quantile of values. Then, the six most frequent *k*-mers were set as important features. The corresponding regression coefficients for the 6 features, *γ*_*j*_ (*j* = 1, …, 6), were simulated from *c*_0_×Uniform (-1, 1). We considered *c*_0_ = 1, 3, 5 for the binary outcome, and *c*_0_ = 1 for the continuous outcome. Given *R*_*i*_ (*i* = 1, …, *n*), we calculated the *n* × *n* TCRhom matrix *K* (Liu et al., 2022). The homology matrix *K* was constructed based on the BLOSUM62 or the PAM250. The *i*th column of *K* represents the similarities between the *i*th individual and the other individuals. Then, in line with Sun et al. (2013), we simulated the hidden effects *h* (*R*_*i*_) ∼ *N* (0, *τK*), where *τ* is a scale factor. We set *τ* to 5 in our simulations. A low-rank approximation via eigen-decomposition was used to ensure that the homology matrix *K* is positive semi-definite. For the binary outcome, the proportion of cases was between 0.3 and 0.7 for the analyzed datasets. For each replicate, we simulated 500 training samples and 500 testing samples. We replicated 500 times for each parameter setting. For performance evaluation, we used classification error and area under the ROC curve (AUC) for the binary outcome, and the mean squared error (MSE) for the continuous outcome.

We compared the proposed TCRpred with potential alternative approaches: Basic-GLM, tcrLASSO, tcrRidge, and DeepTCR. Since the DeepTCR does not consider adjusting-covariates *X*, we first simulated data without adjusting-covariates to compare the considered approaches. For the Basic-GLM, we fitted a GLM model with an intercept. The true underlying value of *k* was used to extract the feature matrix *Z*. For the tcrLASSO and tcrRidge, we conducted a screening on the extracted features *Z* to obtain the top *n*/(2 log  *n*) *k*-mers, and then fit a regularized regression (via either LASSO or Ridge) for the top *k*-mers. For TCRpred, depending on whether *K* was based on BLOSUM62 or PAM250, this approach yielded two versions, TCRpred_B and TCRpred_P. Our simulation results are shown in Supplementary Tables S1, 2, and it can be seen that the proposed TCRpred achieved the highest prediction accuracy among the compared approaches.

Next, we simulated data that included adjusting-covariates, and then compared TCRpred with Basic-GLM, tcrLASSO, and tcrRidge by incorporating *X*. The DeepTCR was omitted because it does not accommodate adjusting covariates. The results are shown in Tables 1-4 for the binary outcome, and in Table 5 for the continuous outcome. TCRpred tends to have a better prediction performance in terms of lower classification errors and higher AUC than the compared approaches. When the data were simulated based on BLOSUM62, the TCRpred_P still performed well. Similarly, when the data were simulated based on PAM250, the TCRpred_B also performed well. This indicated that TCRpred was robust to the choice of the substitution matrices. We also simulated data with *τ* = 8, and the results showed a similar pattern (Supplementary Tables S3–7). In order to examine the influence of choice of *k* in model fitting on the performance of the considered methods, we used *k* = 2, 4 in method application for the binary outcome when 3-mers were employed in data generation. The results showed that the proposed methods still performed well even when *k* was misspecified (Supplementary Tables S8, 9).

**TABLE 1:** Classification error (C.Err) and AUC for the binary outcome. Data were simulated based on 3-mers (*k* = 3) and BLOSUM62.

	*c*_0_ = 1		*c*_0_ = 3		*c*_0_ = 5	
	C.Err	AUC	C.Err	AUC	C.Err	AUC
Basic-GLM	0.355	0.655	0.371	0.628	0.380	0.613
tcrRidge	0.342	0.678	0.333	0.696	0.328	0.708
tcrLASSO	0.345	0.674	0.329	0.700	0.321	0.718
TCRpred_B	0.321	0.715	0.307	0.739	0.297	0.755
TCRpred_P	0.324	0.708	0.310	0.733	0.302	0.748

**TABLE 2:** Classification error (C.Err) and AUC for the binary outcome. Data were simulated based on 4-mers (*k* = 4) and BLOSUM62.

	*c*_0_ = 1		*c*_0_ = 3		*c*_0_ = 5	
	C.Err	AUC	C.Err	AUC	C.Err	AUC
Basic-GLM	0.356	0.655	0.366	0.632	0.375	0.614
tcrRidge	0.343	0.678	0.325	0.703	0.318	0.719
tcrLASSO	0.343	0.677	0.317	0.715	0.305	0.736
TCRpred_B	0.319	0.717	0.296	0.753	0.284	0.772
TCRpred_P	0.323	0.711	0.300	0.745	0.287	0.765

**TABLE 3:** Classification error (C.Err) and AUC for the binary outcome. Data were simulated based on 3-mers (*k* = 3) and PAM250.

	*c*_0_ = 1		*c*_0_ = 3		*c*_0_ = 5	
	C.Err	AUC	C.Err	AUC	C.Err	AUC
Basic-GLM	0.353	0.659	0.370	0.633	0.380	0.615
tcrRidge	0.341	0.678	0.332	0.699	0.327	0.710
tcrLASSO	0.344	0.675	0.330	0.703	0.321	0.719
TCRpred_B	0.324	0.708	0.311	0.734	0.300	0.751
TCRpred_P	0.320	0.716	0.309	0.739	0.298	0.754

**TABLE 4:** Classification error (C.Err) and AUC for the binary outcome. Data were simulated based on 4-mers (*k* = 4) and PAM250.

	*c*_0_ = 1		*c*_0_ = 3		*c*_0_ = 5	
	C.Err	AUC	C.Err	AUC	C.Err	AUC
Basic-GLM	0.354	0.659	0.368	0.634	0.373	0.618
tcrRidge	0.341	0.681	0.328	0.705	0.316	0.720
tcrLASSO	0.341	0.681	0.320	0.716	0.304	0.738
TCRpred_B	0.321	0.713	0.302	0.746	0.287	0.767
TCRpred_P	0.317	0.721	0.298	0.750	0.285	0.770

**TABLE 5:** Mean squared error for the continuous outcome. Data were simulated based on BLOSUM62 (left panel) or PAM250 (right panel).

	BLOSUM62			PAM250		
	*k* = 3	*k* = 4	*k* = 5	*k* = 3	*k* = 4	*k* = 5
Basic-GLM	8.077	8.163	8.166	7.636	7.768	7.857
tcrRidge	7.129	7.343	7.298	6.711	6.917	6.719
tcrLASSO	6.913	6.513	6.196	6.434	6.237	5.963
TCRpred_B	5.426	4.879	4.406	5.503	5.067	4.636
TCRpred_P	5.814	5.294	4.827	5.045	4.631	4.230

## 4 Real data analysis

Lung cancer is the leading cause of cancer-associated death, and non-small cell lung cancer (NSCLC) accounts for approximately 85% of total lung cancer cases (Blandin Knight et al., 2017). Both lung squamous cell carcinoma (LUSC) and lung adenocarcinoma (LUAD) are common subtypes of NSCLC. Evaluating prediction errors requires large sample sizes. Since each of the two datasets has a limited sample size, we used LUSC as the training dataset and LUAD as the testing dataset to evaluate the proposed approach.

We obtained TCR *β*-chain’s CDR3 sequences of LUSC and LUAD by following the same processing and filtering procedure described in Section 3. The details of data processing were given in Supplementary Material S5. We focused on stage I patients because the immune profiles of early stage patients were less likely to be altered by clinical treatments. Following Liu et al. (2022), we dichotomized the overall survival (OS) time into short/long-term survival based on the median survival time in the LUSC and the LUAD data, respectively. We wish to compare the performance of the considered models on the classification of the survival status. The Basic-GLM model included age, gender and Shannon entropy. We adjusted for age, gender and Shannon entropy for tcrLASSO, tcrRidge, and TCRpred. After removing individuals with missing adjusting covariates, 78 and 65 individuals remained in the training and the testing datasets, respectively. The Shannon entropy was computed as $-\sum_{j=1}^{m_{i}}q_{i,j}\log q_{i,j}$ , where $q_{i,j}=w_{i,j}/\sum_{j=1}^{m_{i}}w_{i,j}$ and *w*_*i*,*j*_ is the abundance of the *j*th unique amino acid sequence in the TCR repertoire *R*_*i*_.

We considered *k* = 3 for constructing the extracted-feature matrix *Z*. Each column in *Z* was scaled by the 0.75 quantile of the non-zero entries in the corresponding column. PAM250 was used to construct the homology matrix for TCRpred. For DeepTCR, the V(D)J gene usages were included and the default setting was used. The results of the prediction performance for the compared methods were included in Table 6. Our results showed that the TCRpred had the lowest classification error and highest AUC among these methods. Compared to the tcrLASSO and tcrRidge, the TCRpred additionally considered the TCR-repertoire homology, which harnesses the effects of the hidden features to improve the prediction performance.

**TABLE 6:** Classification error and AUC in TCGA’s LUAD data.

	Basic-GLM	tcrLASSO	tcrRidge	DeepTCR	TCRpred
Classif. Err	0.492	0.431	0.446	0.492	0.400
AUC	0.501	0.625	0.614	0.555	0.661

**FIGURE 2:**
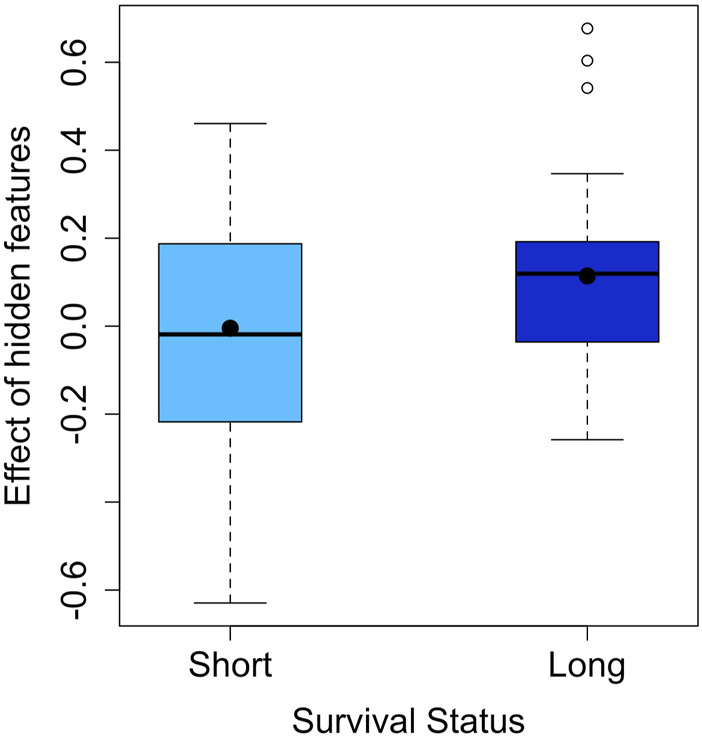
Effect of hidden features in the LUAD dataset (the means are indicated by dots).

**FIGURE 3:**
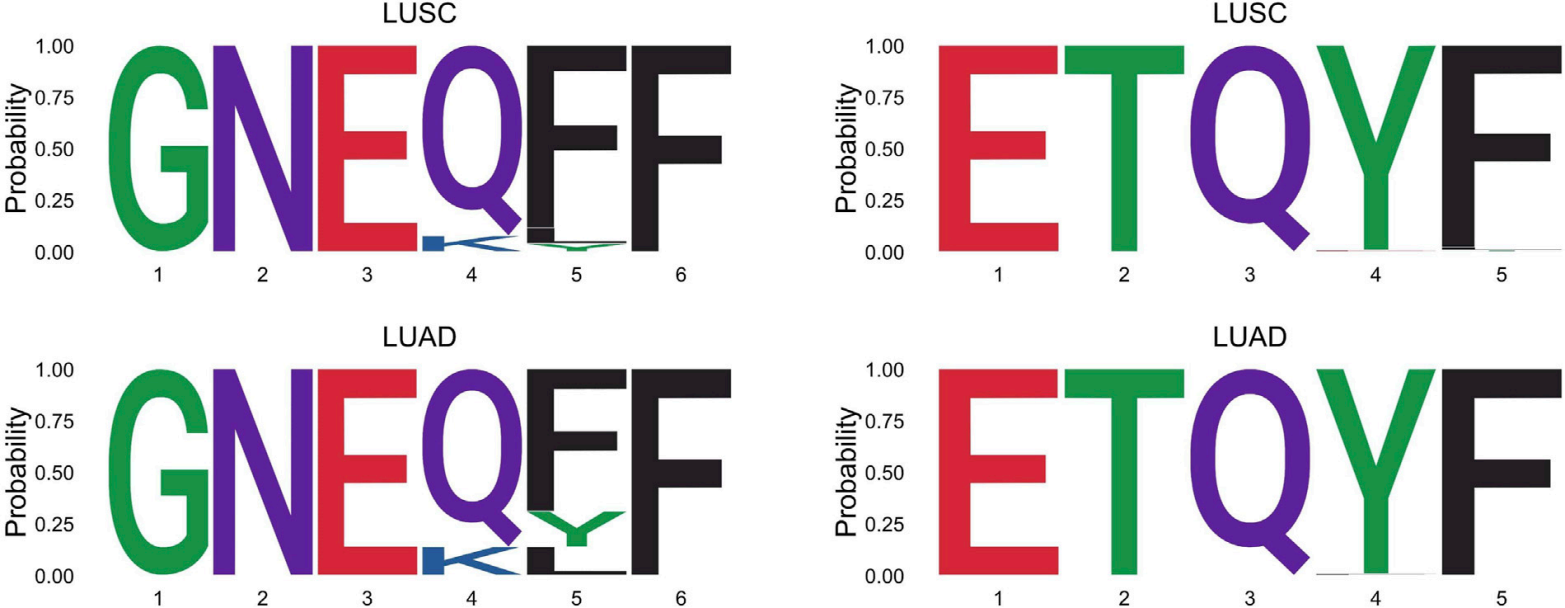
Longer motifs that harbor the GNE or ETQ in the LUSC and LUAD datasets.

The effect of the hidden features for the LUAD dataset is shown in Figure 2. The two survival groups appeared to have different means in their effects of the hidden features. Specifically, for the short survival group, the mean for the effects of the hidden features is close to 0, while for the long survival group, the counterpart is 0.114. For the extracted features, the TCRpred approach identified 7 TCR-sequence features, GNE, ETQ, AGG, GGR, GDT, RYN, and PDR. The tcrLASSO also identified the same set of 3-mers as the TCRpred. The estimated regression coefficients for these 3-mers in the TCRpred model were included in Supplementary Table S10. Further analysis indicated that the GNE was often harbored in the longer motif GNEQFF, and the ETQ was often included in the motif ETQYF (Figure 3). The motif GNEQFF belongs to the T cell receptor beta joining 2-1 (TRBJ2-1) segment (Demarest, 1996). The TRBJ2-1 segment is enriched in lymphoid tissue (Farmanbar et al., 2019) which is closely related to tumor metastasis in resected NSCLC (Rakaee et al., 2021). The motif ETQYF belongs to the TRBJ2-5 segment (Demarest, 1996) which plays potential prognostic roles in predicting postoperative recurrence of NSCLC (Song et al., 2020). It will be interesting to study the potential antigen targets for these motifs, i.e., GNEQFF and ETQYF. Further experimental studies are needed to shed light on the functional importance of the identified *k*-mers and motifs.

## 5 Discussion

We have developed an approach, TCRpred, for incorporating TCR repertoire data for predicting clinical outcomes. Our approach harnesses information from both extracted features and hidden features in the TCR repertoire, and is applicable to both binary and continuous outcomes. With TCR profiling being increasingly used in diagnosis and monitoring of cancer patients, our proposed approach provides a powerful tool for assessing patients’ disease risks and informing decision making in clinical treatment.

It is worth to note that the problem of predicting antigen-cognate for TCR sequences (Hudson et al., 2023) is different from the problem of using TCR repertoire to predict clinical outcome. For the former problem, the predictor is a single TCR sequence, while for the latter, the predictors are a large set of TCR sequences with different lengths and compositions, along with demographic and clinical variables. The latter problem requires aggregating information across different TCR sequences and further integrate genetic and clinical variables for predicting the outcome. Nevertheless, both problems are highly challenging, and more research efforts are needed to study these important problems.

In our analysis, the extracted feature matrix was constructed based on an exhaustive search of amino acid *k*-mers in the studied TCR repertoire. Such an agnostic approach ensures that every possible *k*-mers is interrogated, but on the other hand, the exhaustive search brings in the high-dimensionality issue. The dimension of the extracted features increases exponentially with *k*, posing tremendous challenges to data analysis when *k* is large. To overcome such challenges, a possible strategy is to utilize prior biological knowledge to narrow down the scope of *k*-mers being searched, and then focus on a smaller set of *k*-mers for risk prediction. Multiple databases have been built to include both antigen information and the corresponding TCR sequences, such as the VDJdb (Bagaev et al., 2020). While many of the collections in these databases are derived from infectious disease studies, collections on tumor antigens and their TCR sequences are expected to grow significantly in the coming years. It will be highly meaningful to explore the use of such databases for improving the power of risk prediction.

TCR repertoire involves dynamic changes along disease progression and clinical treatment, and some studies have been designed to monitor the TCR repertoire at multiple time points (Öjlert et al., 2021). Such studies capture not only the immuno profiles at different stages, but also the shift of certain sub-populations of the T cells which may be critical for evaluating treatment responses. On the other hand, the involvement of longitudinal data adds one more layer of complexity to the TCR repertoire analysis, and how to effectively analyze such data remains to be investigated.

To conclude, the proposed method, TCRpred, can be used for clinical outcome prediction by harnessing both the compositions and the sequence information of the TCR repertoire. Our simulation studies showed that the TCRpred outperformed the compared alternative approaches under various parameter settings. In real data analysis, the proposed method performed well and identified a group of *k*-mers that are potentially related to the survival status of lung cancer patients. Overall, the TCRpred adds a useful tool to the existing toolbox for the analysis of TCR repertoire.

## Data Availability

Clinical data can be accessed at https://xenabrowser.net/datapages/. The TCR data were extracted from RNASeq data, which can be accessed at https://portal.gdc.cancer.gov/ with approval from GDC.
