# Supplementary material for: TCRpred: incorporating T-cell receptor repertoire for clinical outcome prediction
Source: Front Genet. 2024 Mar 13;15:1345559. doi: 10.3389/fgene.2024.1345559 (PMC10965803; doi:10.3389/fgene.2024.1345559)
Supplement: Supplementary file 1 [file DataSheet1.pdf]

Supplementary Material

1 DEMONSTRATION OF EXTRACTING K-MERS ($K = 2$)

Subj. ID	Nucleotide Sequence	Amino Acid Sequence	Abundance	V segment	⋯
1	TGTGCCACCAGCCCTAGGAATCAGCCCCAGCATTTT	CATSPRNQPQHF	9	TRBV15*01	⋯
1	TGCAGTGCCCCGGGACAGGGGAAAGAGACCCAGTACTTC	CSAPGQGKETQYF	1	TRBV20-1*01	⋯
1	TGTGCCAGCAGCTTCTTCGCATACAATGAGCAGTTCTTC	CASSFFAYNEQFF	4	TRBV11-1*01	⋯
2	TGCAGTGCTACTAGCGGGAGTACTTCCTACGAGCAGTACTTC	CSATSGSTSYEQYF	5	TRBV20-1*01	⋯
2	TGCAGCGTTGAAGATGCAGGGGGCGGTGAGCAGTTCTTC	CSVEDAGGGEQFF	1	TRBV29-1*01	⋯
2	TGTGCCAGCGGGACAGGCGGGTCACCCCTCCACTTT	CASGTGGSPLHF	2	TRBV19*01	⋯
2	TGTGCCAGCAGCTGTGCCGGTTGTGCCTATCATTATTTT	CASSCAGCAYHYF	5	TRBV12-5*01	⋯
3	TGTGCCACCAGTGCCCCCTACACTGAAGCTTTCTTT	CATSAPYTEAFF	3	TRBV15*02	⋯
3	TGTGCCAGCAGCAACGTTCACACTGAAGCTTTCTTT	CASSNVHTEAFF	12	TRBV5-5*01	⋯
⋮	TGTGCCAGCTCACCCAGCACAGATACGCAGTATTTT	CASSPSTDTQYF	4	TRBV18*01	⋯
	⋮	⋮	⋮	⋮	⋯

Example of data structure of the TCR β chain's CDR3 region

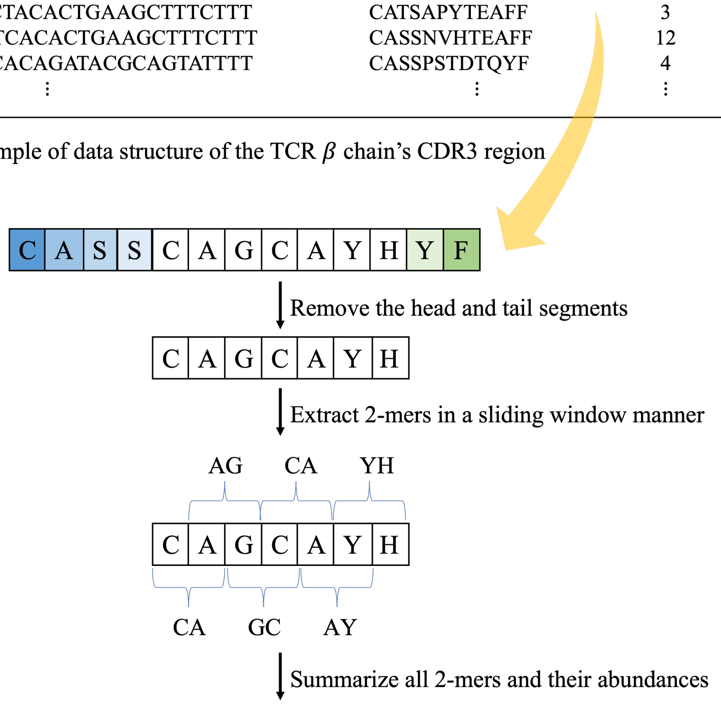

Polypeptide	CA	AG	GC	AY	YH
Frequency	10	5	5	5	5

Figure S1. An example of extracting 2-mers from an amino acid sequence. The head and tail segments (CASS and YF) were removed before extracting features due to their high conservation across TCR sequences. The frequencies of each 2-mer are then aggregated across all TCR sequences of a person's TCR repertoire for regression analysis.

2 ADDITIONAL SIMULATION STUDY I

In this simulation study, we compared five methods, Basic-GLM, tcrLASSO, tcrRidge, DeepTCR TCRpred_B and TCRpred_P, under different parameter settings. Since DeepTCR does not accommodate adjusting covariates, the data were generated without adjusting covariates.

We focused on the binary outcome. We considered $k = 3$ (i.e., 3-mers) and let the six most frequent 3-mers be important features. The corresponding coefficients γ_j follow a c_0Uniform(-1, 1) for $j = 1, \cdots, 6$. We considered $c_0 = 1, 3$, and 5. The scaling parameter for K were set as $\tau = 5$ and 8. The homology matrix K for data simulation was constructed based on the substitution matrix of BLOSUM62. The proportion of cases was between 0.3 and 0.7 for the analyzed datasets. For each replicate, we simulated 500 individuals for parameter estimation and 500 individuals for prediction evaluation. We replicated 500 times for each parameter setting. For DeepTCR, the V(D)J gene segment usages were included and the default argument setting was used, and the 500 training individuals were split into two sets: 350 for training and 150 for validation. For performance evaluation, we used classification error (C.Err) and area under the ROC curve (AUC) over 500 replicates. The results are shown in Supplementary Tables S1 and S2. The proposed TCRpred outperforms the compared approaches, including Basic-GLM, tcrRidge, tcrLASSO, and DeepTCR. The Basic-GLM does not account for the TCR information, while the tcrRidge and tcrLASSO do not accommodate the TCR homology information among TCR repertoires. The DeepTCR encodes each TCR sequence by an embedding vector, and then aggregates multiple TCR sequences into a single numerical vector to represent a TCR repertoire. This aggregation process is likely to cause a loss of information, which may partly explains its less accurate prediction performance.

Table S1. Classification error (C.Err) and AUC for the binary outcome when $\tau = 5$

	$c_0 = 1$		$c_0 = 3$		$c_0 = 5$	
	C.Err	AUC	C.Err	AUC	C.Err	AUC
Basic-GLM	0.406	0.500	0.411	0.500	0.413	0.500
tcrRidge	0.404	0.553	0.388	0.592	0.372	0.630
tcrLASSO	0.406	0.553	0.380	0.610	0.354	0.661
DeepTCR	0.431	0.545	0.415	0.561	0.401	0.579
TCRpred_B	0.371	0.635	0.348	0.673	0.326	0.711
TCRpred_P	0.376	0.625	0.353	0.664	0.329	0.706

Table S2. Classification error (C.Err) and AUC for the binary outcome when $\tau = 8$

	$c_0 = 1$		$c_0 = 3$		$c_0 = 5$	
	C.Err	AUC	C.Err	AUC	C.Err	AUC
Basic-GLM	0.405	0.500	0.409	0.500	0.411	0.500
tcrRidge	0.401	0.556	0.389	0.588	0.375	0.621
tcrLASSO	0.403	0.556	0.383	0.602	0.361	0.647
DeepTCR	0.427	0.550	0.415	0.563	0.402	0.578
TCRpred_B	0.356	0.658	0.344	0.681	0.325	0.713
TCRpred_P	0.363	0.645	0.350	0.672	0.330	0.705

3 ADDITIONAL SIMULATION STUDY II

We conducted stimulation study for $\tau = 8$. Other parameter settings were in line with those in the main text. As to the results, we observed a similar pattern to that in the main text where $\tau = 5$.

Table S3. Classification error (C.Err) and AUC for the binary outcome. Data were simulated based on 3-mers ($k = 3$) and BLOSUM62.

	$c_0 = 1$		$c_0 = 3$		$c_0 = 5$	
	C.Err	AUC	C.Err	AUC	C.Err	AUC
Basic-GLM	0.366	0.639	0.380	0.618	0.388	0.604
tcrRidge	0.355	0.657	0.346	0.681	0.339	0.694
tcrLASSO	0.357	0.654	0.343	0.685	0.333	0.703
TCRpred_B	0.323	0.713	0.311	0.738	0.302	0.751
TCRpred_P	0.328	0.704	0.316	0.730	0.308	0.743

Table S4. Classification error (C.Err) and AUC for the binary outcome. Data were simulated based on 4-mers ($k = 4$) and BLOSUM62.

	$c_0 = 1$		$c_0 = 3$		$c_0 = 5$	
	C.Err	AUC	C.Err	AUC	C.Err	AUC
Basic-GLM	0.367	0.639	0.377	0.618	0.383	0.606
tcrRidge	0.354	0.661	0.338	0.688	0.327	0.705
tcrLASSO	0.354	0.661	0.330	0.699	0.316	0.722
TCRpred_B	0.320	0.719	0.300	0.751	0.286	0.771
TCRpred_P	0.326	0.709	0.305	0.743	0.290	0.763

Table S5. Classification error (C.Err) and AUC for the binary outcome. Data were simulated based on 3-mers ($k = 3$) and PAM250.

	$c_0 = 1$		$c_0 = 3$		$c_0 = 5$	
	C.Err	AUC	C.Err	AUC	C.Err	AUC
Basic-GLM	0.364	0.643	0.378	0.622	0.386	0.607
tcrRidge	0.353	0.661	0.343	0.684	0.336	0.697
tcrLASSO	0.355	0.658	0.340	0.687	0.331	0.706
TCRpred_B	0.325	0.707	0.313	0.732	0.302	0.749
TCRpred_P	0.319	0.718	0.309	0.739	0.299	0.755

Table S6. Classification error (C.Err) and AUC for the binary outcome. Data were simulated based on 4-mers ($k = 4$) and PAM250.

	$c_0 = 1$		$c_0 = 3$		$c_0 = 5$	
	C.Err	AUC	C.Err	AUC	C.Err	AUC
Basic-GLM	0.365	0.642	0.376	0.622	0.382	0.607
tcrRidge	0.351	0.664	0.338	0.690	0.326	0.708
tcrLASSO	0.351	0.663	0.330	0.702	0.315	0.725
TCRpred_B	0.322	0.713	0.305	0.744	0.289	0.766
TCRpred_P	0.317	0.724	0.301	0.751	0.286	0.771

Table S7. MSE for the continuous outcome. Data were simulated based on BLOSUM62 (left panel) or PAM250 (right panel).

	BLOSUM62			PAM250		
	$k = 3$	$k = 4$	$k = 5$	$k = 3$	$k = 4$	$k = 5$
Basic-GLM	10.241	10.338	10.395	9.609	9.735	9.822
tcrRidge	9.317	9.664	9.682	8.764	9.097	8.931
tcrLASSO	9.198	8.898	8.737	8.625	8.423	8.134
TCRpred_B	6.436	5.976	5.670	6.678	6.318	5.832
TCRpred_P	7.114	6.655	6.353	5.965	5.634	5.180

4 ADDITIONAL SIMULATION STUDY III

We conducted stimulation studies for the binary outcome. In data generation, we considered $k = 3$, $\tau = 5$ and subtitution matrix BLOSUM62. Other parameter settings were in line with those in the main text. In model fitting, we considered $k = 2, 4$. As to the results, we observed that the proposed methods still performed well even when k was misspecified.

Table S8. Classification error (C.Err) and AUC when 2-mers were used for model fitting.

	$c_0 = 1$		$c_0 = 3$		$c_0 = 5$	
	C.Err	AUC	C.Err	AUC	C.Err	AUC
tcrRidge	0.341	0.680	0.329	0.701	0.322	0.716
tcrLASSO	0.342	0.679	0.319	0.714	0.307	0.736
TCRpred_B	0.317	0.721	0.298	0.749	0.285	0.768
TCRpred_P	0.321	0.713	0.301	0.743	0.290	0.761

Table S9. Classification error (C.Err) and AUC when 4-mers were used for model fitting.

	$c_0 = 1$		$c_0 = 3$		$c_0 = 5$	
	C.Err	AUC	C.Err	AUC	C.Err	AUC
tcrRidge	0.341	0.680	0.331	0.698	0.327	0.710
tcrLASSO	0.342	0.678	0.330	0.701	0.323	0.715
TCRpred_B	0.318	0.719	0.308	0.739	0.301	0.751
TCRpred_P	0.322	0.712	0.312	0.732	0.305	0.744

5 SUPPLEMENTARY MATERIAL FOR REAL DATA ANALYSIS

We obtained TCR β-chain's CDR3 sequences of LUSC and LUAD by following the same processing and filtering procedure described in Section 3. We extracted TCR beta-chain's CDR3 sequences from TCGA's RNA-Seq data. We removed TCR sequences that had abundance equal to 1 or contained abnormal amino acid letters. Individuals with a single TCR sequence were excluded. The number of unique TCR sequences in each subject, named as u_i, ranges from 2 to 91. The distribution of u_i is shown in Supplementary Figure S2. Individuals whose diversities are relatively low (with the number of unique sequences $< 30\%$ quantile of the u_i) were omitted from the analysis. The survival time and clinical features were obtained from TCGA. We dichotomized the overall survival (OS) time into short/long-term survival (0 for short, and 1 for long) based on the median survival time in the LUSC and the LUAD data, respectively.

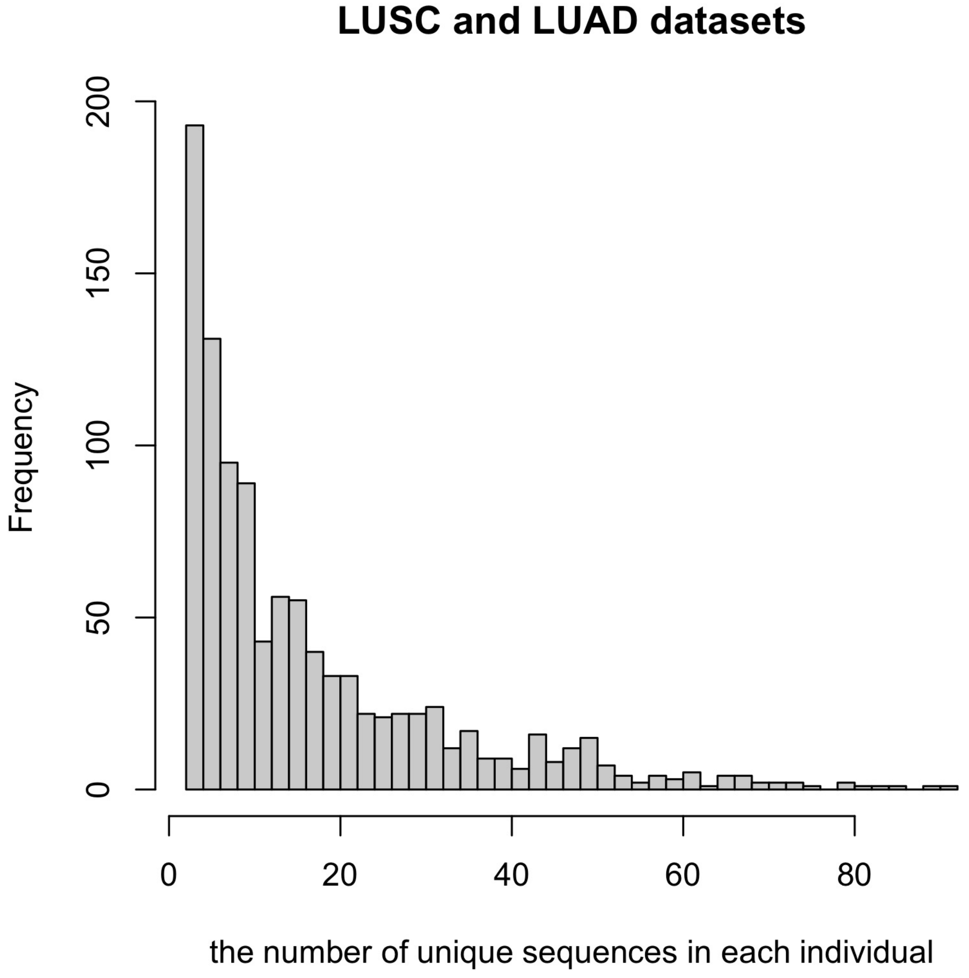

Figure S2. Histogram of the number of unique amino acid sequences in each individual for pooled LUSC and LUAD dataset.

6 COEFFICIENT ESTIMATES OF THE EXTRACTED FEATURES BY THE TCRPRED APPROACH

Table S10. Coefficient estimates of the extracted features by the TCRpred

	PDR	RYN	GNE	AGG	GGR	GDT	ETQ
Estimates	0.990	0.987	0.390	0.133	0.117	0.047	0.039
